# From symptom tracking to prevention – A transformer-based dynamic model for predicting mild cognitive impairment risk in older adults with depression: A longitudinal study based on CHARLS and CLHLS

**DOI:** 10.1097/MD.0000000000048653

**Published:** 2026-05-08

**Authors:** Yirui Chen, Siyi Kong, Tianyun Wang, Junxing Chen, Kai Ma, Junzhi Zhang, Ye Zhang, Mengyang Wang

**Affiliations:** a College of Public Health, Tianjin University of Traditional Chinese Medicine, Tianjin, China; b College of Nursing, Guangxi University of Chinese Medicine, Guangxi, China; c College of Culture and Health Communication, Tianjin University of Traditional Chinese Medicine, Tianjin, China; d Centre for Epidemiology and Biostatistics, Melbourne School of Population and Global Health, University of Melbourne, Melbourne, Australia.

**Keywords:** depression, mild cognitive impairment, time series, transformer

## Abstract

Depression in older adults is closely associated with an increased risk of mild cognitive impairment (MCI), yet existing prediction models often rely on cross-sectional data and fail to capture temporal changes in depressive symptoms. This study aimed to develop and validate a transformer-based dynamic prediction model for MCI risk in older adults with depression using longitudinal data. Data were obtained from the China Health and Retirement Longitudinal Study. A total of 2119 older adults with depressive symptoms were included. A sliding-window time-series framework was constructed using longitudinal follow-up data, and 394 key features were retained after feature screening and missing-data processing. An optimized transformer model incorporating dynamic positional encoding, multi-head self-attention, and gated feedforward networks was developed to model temporal associations between depressive symptom trajectories and subsequent MCI risk. Model performance was compared with that of Extreme Gradient Boosting and support vector machine. External validation was further conducted using data from the Chinese Longitudinal Healthy Longevity Survey. On the test set, the optimized transformer model achieved an accuracy of 0.816 and an area under the receiver operating characteristic curve (AUC) of 0.851, outperforming Extreme Gradient Boosting (AUC = 0.807) and support vector machine (AUC = 0.776). The transformer model also showed superior precision (0.892), specificity (0.841), sensitivity (0.801), and F1 score (0.844), indicating a stronger ability to identify high-risk individuals and capture long-term temporal dependencies in depressive symptom patterns. In external validation using the Chinese Longitudinal Healthy Longevity Survey dataset, the model maintained good generalizability, with an F1 score of 0.783 and an AUC of 0.821. The proposed transformer-based dynamic model demonstrated strong predictive performance and generalizability for identifying MCI risk in older adults with depression. By incorporating longitudinal depressive symptom trajectories, this approach provides a potentially useful tool for early screening, risk stratification, and preventive intervention in aging populations.

## 1. Introduction

Mild cognitive impairment (MCI), recognized as an intermediate state of cognitive dysfunction, is academically positioned as a transitional phase between normal aging and dementia.^[1]^ This condition exhibits 3 progression trajectories: conversion to dementia, persistent cognitive impairment, or recovery to normal cognition, with the highest conversion rate to dementia reaching 60% to 65%.^[2]^ Depression, characterized by persistent low mood and behavioral inhibition,^[3]^ affects approximately 5% of adults globally according to WHO data. While severe cases carry self-harm risks, stepped care protocols demonstrate proven efficacy across depression severity levels.^[4]^ Against the backdrop of population aging, the incidence of geriatric depression continues to rise,^[5]^ with epidemiological surveys revealing a lifetime prevalence of 3.4%, with 50% of recurrent cases accompanied by cognitive impairment.^[6]^ Notably, elderly depression patients exhibit 2.4 to 3.1 times higher risk of developing MCI compared to healthy populations,^[7]^ and 32% of existing MCI cases present comorbid depressive symptoms.^[8]^ Joyce Y C Chan team confirmed that depression significantly elevates dementia and MCI risks, with antidepressant use potentially exacerbating this association.^[9]^ However, clinical practice faces dual challenges: overlapping symptoms between geriatric depression and cognitive decline hinder early detection,^[10]^ while existing research primarily focuses on correlation validation^[11]^ with predictive models relying on cross-sectional data,^[12]^ lacking temporal analysis of depressive symptom dynamics and cognitive trajectory associations.

Depression significantly impacts mental activities in older adults, often manifesting as reversible cognitive impairment, clinically termed pseudodementia syndrome or depression-related cognitive dysfunction.^[13]^ Studies show MCI patients with comorbid depression face significantly higher dementia conversion risks than the general population.^[14]^ Current clinical interventions face dilemmas: cholinesterase inhibitors (e.g., donepezil and galantamine),^[15]^ antioxidants (vitamin E),^[16]^ NSAIDs (celecoxib, rofecoxib, and naproxen),^[17]^ and cognitive enhancers (lenacapavir)^[18]^ show limited efficacy in MCI treatment, while nutritional interventions like folic acid yield minimal benefits.^[19]^ In contrast, the relatively mature depression treatment framework offers standardized protocols for mild,^[20]^ moderate,^[21]^ and severe cases,^[22]^ creating feasible pathways for MCI prevention through depression management. The high comorbidity rate of depression in MCI patients and their bidirectional association highlight the critical role of dynamically tracking depressive symptom trajectories for MCI risk stratification, underscoring the need for early warning systems based on depression dynamics.

Current clinical prediction models exhibit notable limitations in dynamic risk assessment: Traditional Cox proportional hazards models, constrained by time-invariant covariate assumptions, fail to capture dynamic associations between depressive fluctuations and cognitive decline.^[23]^ While static models like random forests integrate multi-source data, their temporal feature extraction relies on manual design strategies,^[24]^ resulting in lost long-term dependencies in temporal dimensions.^[25]^ Notably, transformer architectures – with advantages in self-attention mechanisms and temporal sequence modeling – have demonstrated breakthrough applications in medical prediction,^[26]^ yet remain unexplored for dynamic risk prediction of MCI secondary to geriatric depression. This study, leveraging the China Health and Retirement Longitudinal Study (CHARLS) database,^[27]^ constructs a transformer-based dynamic depression trajectory prediction model by integrating multidimensional longitudinal data (depression scale scores, cognitive assessments, and physiological parameters) from elderly depression patients. This innovative approach aims to enable precise quantitative assessment of secondary MCI risks, providing decision support tools for early clinical intervention and potentially disrupting the malignant transformation pathway from geriatric depression to cognitive impairment. The research holds significant practical value for enhancing healthcare in aging societies.

## 2. Methods

### 2.1. Depression assessment

This study employed the abbreviated 10-item Center for Epidemiologic Studies Depression Scale (CESD-10) to evaluate depressive symptoms in participants. The scale has demonstrated excellent psychometric properties for detecting depression in Chinese adults.^[28]^ The CESD-10 comprises 10 items scored on a 0 to 3 scale, reflecting responses ranging from “none” to “nearly every day.” Total scores range from 0 to 30, with higher scores indicating greater severity of depressive symptoms. For simplicity, the term “depression” encompasses varying degrees of symptom severity.^[29]^ Individuals scoring ≥10 were classified as having depressive symptoms, while those scoring <10 were defined as normal.

### 2.2. Cognitive function measurement

A multidimensional neuropsychological assessment framework was used to quantify cognitive function across 2 core dimensions: episodic memory and global mental status, with total scores ranging from 0 to 31 (higher scores indicate better cognitive reserve).^[30]^

Episodic memory: Assessed via standardized word recall tasks (score range: 0–20), including immediate recall and delayed recall subtasks. Participants were presented with a standardized list of 10 Chinese words and asked to freely recall them immediately (immediate recall, 0–10 points). After an interval of other cognitive tests (to eliminate short-term memory interference), participants performed a delayed recall task (delayed recall, 0–10 points). Each accurately recalled word scored 1 point, with total scores reflecting encoding, storage, and retrieval efficiency.

Global mental status: Evaluated using a modified Telephone Interview of Cognitive Status combined with a figure-copying task (total score range: 0–11). This included:

1. Temporal orientation (0–5 points): Assessed via 5 hierarchical time-anchoring questions (current year, month, date, day of the week, and season), with 1 point awarded for each correct answer.
2. Calculation ability (0–5 points): Tested through a serial subtraction task (e.g., subtracting 7 from 100 consecutively 5 times), with 1 point per correct step.
3. Visuospatial function (0–1 point): Assessed via a pentagon-crossing replication task, with 1 point awarded for accurate topological reproduction.^[31]^

Given the lack of unified diagnostic criteria for MCI, this study adopted an age-associated cognitive decline model to establish an objective diagnostic framework adjusted for demographic characteristics.^[32]^ Specifically, participants aged ≥60 years were stratified into 5-year intervals (e.g., 60–64 and 65–69). Within each age stratum, individuals with total cognitive scores below the mean −1 standard deviation were classified as MCI. This approach avoids overreliance on subjective reports inherent in traditional Petersen criteria while enhancing diagnostic objectivity through standardized statistical thresholds.

### 2.3. Data sample composition

Data were sourced from the CHARLS database (http://charls.pku.edu.cn), a nationally representative platform for middle-aged and elderly populations. The database employs multistage stratified sampling and includes multidimensional structured data on sociodemographics, chronic disease history, biochemical markers, and mental health assessments. Ethical approval for CHARLS was obtained from the Biomedical Ethics Review Committee of Peking University (IRB00001052-11015), and written informed consent was obtained from all participants. Ethical approval for Chinese Longitudinal Healthy Longevity Survey was obtained from the Ethics Committee of Peking University and Duke University (IRB00001052-13074).

**Figure 1.**
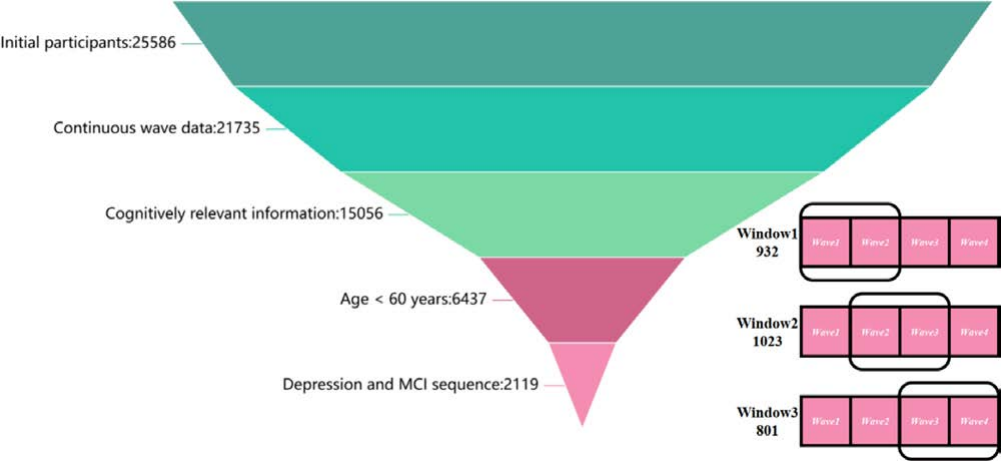
Flowchart of participant selection from the CHARLS database. CHARLS = China Health and Retirement Longitudinal Study, MCI = mild cognitive impairment.

From an initial cohort of 25,586 participants across 4 waves (2011, 2013, 2015, and 2018),^[33]^ exclusion criteria were applied: 3851 participants lacked ≥2 consecutive waves of data; 6679 lacked cognitive assessment data; 8619 were aged <60 years; and 4318 had unclear temporal sequences between depression and MCI onset. The final cohort comprised 2119 participants.^[34]^ For longitudinal data with time-dependent characteristics, a sliding window approach was selected to align with the fixed-length input requirements of transformer architectures and efficiently capture the triggering effect of depression on MCI. Three sliding windows were constructed: Wave 1 → Wave 2, Wave 2 → Wave 3, and Wave 3 → Wave 4, yielding 932, 1023, and 801 samples, respectively. The workflow is illustrated in Figure 1.^[35]^

### 2.4. Feature processing

Feature selection plays a critical role in constructing predictive models for depression-related cognitive frailty in older adults. By systematically screening variables to eliminate redundancy and reduce noise interference, it enhances model generalization. Based on CHARLS database multidimensional tracking data, this study employed a rigorous multi-stage screening framework^[36]^: Exclusion of dependent variables (cognitive assessment metrics) and their derivative composite indicators to avoid circular reasoning; removal of variables with inconsistent measurement tools or definitions across the 4 survey waves (e.g., mixed scale versions); deletion of variables with overall missing rates >20% to control interpolation errors; and elimination of composite indicators with high content overlap with other variables (e.g., total scale scores vs subdomain scores). This process reduced 1217 original indicators to 394 valid features, covering core dimensions including dynamic trajectories of depressive symptoms, preclinical markers of cognitive decline, and confounding factor regulation (sociodemographics, comorbidity indices, and lifestyle),^[37]^ ensuring a comprehensive and reliable feature space.

For residual missing values (overall missing rate ≤ 20%) in the selected features, we adopted a multiple imputation (MI)-based mean imputation strategy following the approach of Kai Ma et al.^[26]^ This method predicts missing value distributions through iterative regression models and substitutes conservative mean values to avoid introducing excessive variance. This preserves data integrity while mitigating interference from outliers on transformer attention mechanisms. Subsequently, a sliding window strategy was applied to segment longitudinal data into temporal slices, capturing MCI triggering risks under depressive symptomatology through overlapping windows.^[38]^ The final causal dataset comprised 2756 sample units, each containing multidimensional features from consecutive time windows, ensuring input sequences align with the transformer model’s requirements for modeling local temporal dependencies.

### 2.5. Model architecture

**Figure 2.**
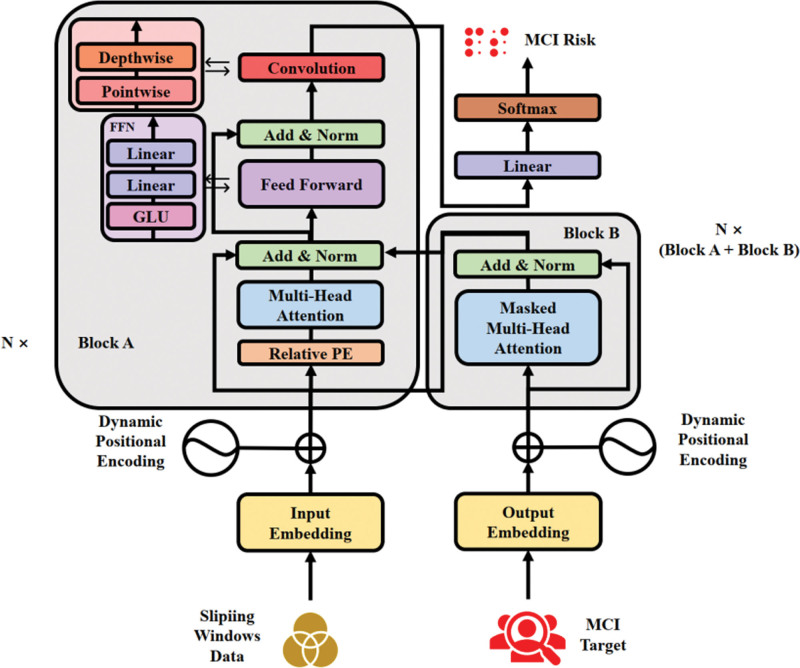
Architecture of the proposed transformer-based model. FFN = feedforward neural network, GLU = gated linear unit, MCI = mild cognitive impairment.

The transformer-based predictive model for depression-induced cognitive decline in older adults integrates technical components including optimized multi-head attention mechanisms, dynamic positional encoding, gated feedforward structures, and adaptive normalization layers. Regularization strategies such as early stopping and dropout were employed to mitigate overfitting risks. The system architecture and configurations are illustrated in Figure 2.

#### 2.5.1. Encoder-decoder framework

A transformer-based encoder-decoder architecture was adopted to model the complex temporal relationships between depressive symptoms and cognitive decline. The encoder encodes input depression symptom sequences into high-dimensional semantic representations, while the decoder predicts future cognitive states through generative methods. Model dimensions were set to 512, with 8 attention heads in multi-head self-attention layers to capture fine-grained features. A depthwise separable convolution module^[39]^ was introduced to enhance representational capacity. This module, placed after feedforward networks in each encoder/decoder sublayer, combines depthwise and pointwise convolutions to extract local features and strengthen nonlinear modeling:


$$\begin{array}{l} Depthwise\;Separable\;Convolution\;\left(x\right)=Pointwise\;Conv\;\left(Depthwise\;Conv\left(x\right)\right) \end{array}$$ (1)

Here *x* is the input feature, Depthwise Conv performs a convolution operation on each input channel individually, while Pointwise Conv fuses the information between the channels through 1 × 1 convolution, allowing the model to capture local features in the sequence more efficiently.

#### 2.5.2. Optimized self-attention mechanism

The self-attention mechanism – a core component of transformer architectures – effectively captures global correlations between elements within sequences. This study employs multi-head attention mechanisms to achieve multi-dimensional feature space analysis by projecting the original input into multiple independent subspaces for parallel computation. To enhance temporal modeling capabilities, each attention head incorporates a relative positional encoding module that quantifies relative distances between elements through learnable positional bias parameters.^[40]^ This strengthens the model’s ability to capture long-range dependencies in sequential data. The mathematical formulation of this mechanism is expressed as:


$$\begin{array}{l} MultiHead\;\left(Q,K,V\right)=\;Concat\;\left(head_{1},head_{2},head_{3},...,head_{h}\right)W^{O} \end{array}$$ (2)

$$\begin{array}{l} head_{i}=\;Attention\;\left(QW_{i}^{Q},KW_{i}^{K},VW_{i}^{V}\right) \end{array}$$ (3)

$$\begin{array}{l} Attention\;\left(Q,K,V\right)=\;softmax\left(\frac{QK^{T}+B}{\sqrt{d_{k}}}\right)V \end{array}$$ (4)

Here Q, *K*, *V* are query, key, and value matrices, $W_{i}^{Q}$, $W_{i}^{K}$, $W_{i}^{V}$ denote weight matrices for the i-th attention head, $W^{O}$ is the output weight matrix, *B* represents relative positional bias, and $d_{k}$ denotes the dimensionality of the attention mechanism.

#### 2.5.3. Dynamic positional encoding

Traditional positional encoding methods (e.g., sine/cosine functions) provide sequence order information but may cause positional ambiguity in long sequences. To address this limitation, this study adopts a dynamic positional encoding mechanism. This approach introduces a learnable positional embedding vector, which is added element-wise to input feature vectors to deliver more precise positional information.^[41]^ The mathematical formulation is expressed as:


$$\begin{array}{l} PE_{\left(pos\right)}=\;LearnableEmbedding\left(pos\right) \end{array}$$ (5)

Here $PE_{\left(pos\right)}$ denotes the dynamic positional encoding for the pos-th position, and LearnableEmbedding () represents a trainable embedding function. This mechanism enables adaptive adjustment of positional encodings to better accommodate varying sequence lengths and feature distributions.

#### 2.5.4. Feedforward neural network (FFN)

The FFN in this study serves as a post-processing unit for self-attention modules, enhancing feature representation through nonlinear mapping. Specifically, the FFN structure comprises 2 fully connected layers with Gaussian error linear unit activation functions. To strengthen nonlinear feature extraction, this study innovatively incorporates a gated linear unit mechanism. This dual-path computational framework enables feature filtering: one path generates weighting coefficients via a sigmoid gate, while the other preserves raw feature information.^[42]^ This dynamic modulation mechanism allows the network to adaptively select critical feature dimensions, thereby enhancing modeling capabilities for complex nonlinear relationships in sequential data. The mathematical formulation is defined as:


$$\begin{array}{l} GLU\left(x\right)=x\sigma \left(Wx+b\right) \end{array}$$ (6)

Here *x* denotes the input vector, *W* and *b* represent weight matrices and bias terms, σ signifies the sigmoid activation function.

#### 2.5.5. Layer normalization and residual connections

In this study, layer normalization and residual connections were integrated into all submodules of the encoder-decoder architecture (including self-attention and feedforward network components) to enhance model robustness, accelerate parameter convergence, and suppress gradient anomalies. Layer normalization stabilizes input distributions across network layers through feature-wise standardization, while residual connections preserve raw input information via shortcut pathways, effectively reducing optimization complexity in deep networks. The mathematical formulation of this dual optimization mechanism is defined as:


$$\begin{array}{l} Output=\;LayerNorm\;\left(x+\;Sublayer\left(x\right)\right) \end{array}$$ (7)

Here *x* denotes the input, Sublayer (*x*) represents the output of the submodule, LayerNorm refers to layer normalization with ϵ = 1e − 12 to ensure numerical stability. To further enhance optimization, an adaptive layer normalization (AdaLN) mechanism was introduced. AdaLN dynamically adjusts normalized outputs through learnable scaling (γ) and shifting (β) parameters, enabling adaptive adaptation to varying feature distributions.^[43]^ The mathematical expression is:


$$\begin{array}{l} AdaLN\left(x\right)=\gamma LayerNorm\left(x\right)+\beta \end{array}$$ (8)

Here γ and β are learnable parameters that modulate the normalized outputs.

#### 2.5.6. Model training and optimization

The model is trained by minimizing the loss function between predicted cognitive states and ground-truth values. The cross-entropy loss function is employed, expressed as:


$$\begin{array}{l} \iota =-\underset{i}{\overset{N}{\sum }}\;\left(y_{i}\log{\hat{y}}_{i}+\left(1-y_{i}\right)\log\left(1-{\hat{y}}_{i}\right)\right) \end{array}$$ (9)

Here *N* denotes sample count, $y_{i}$ represents the ground-truth label for the *i*-th sample, and ${\hat{y}}_{i}$ is the predicted probability.

For parameter optimization, the AdamW algorithm with adaptive learning rate scaling is utilized. This method decouples weight decay mechanisms from parameter update strategies, achieving dual control over optimization through first- and second-moment gradient estimates while introducing an independent weight decay term for parameter regularization.^[44]^ The mathematical formulation is defined as:


$$\begin{array}{l} m_{t}=\beta_{1}m_{t-1}+\left(1-\beta_{1}\right)g_{t} \end{array}$$ (10)

$$\begin{array}{l} v_{t}=\beta_{2}v_{t-1}+\left(1-\beta_{2}\right)g_{t}^{2} \end{array}$$ (11)

$$\begin{array}{l} {\hat{m}}_{t}=\frac{m_{t}}{1-\beta_{1}^{t}} \end{array}$$ (12)

$$\begin{array}{l} {\hat{v}}_{t}=\frac{v_{t}}{1-\beta_{2}^{t}} \end{array}$$ (13)

$$\begin{array}{l} \theta_{t}=\theta_{t-1}-\frac{\alpha }{\sqrt{{\hat{v}}_{t}}+\epsilon }{\hat{m}}_{t}-\lambda \theta_{t-1} \end{array}$$ (14)

Here $m_{t}$ and $v_{t}$ are first- and second-moment estimates of gradients, $\beta_{1}$ and $\beta_{2}$ are decay coefficients, $g_{t}$ is the gradient at current step, $\theta_{t}$ is the model parameters, α is the learning rate, ϵ is a constant used for numerical stabilization, and λ is the weight decay factor. In this study, the learning rate α was set to 3e−4, $\beta_{1}$ was set to 0.9, $\beta_{2}$ was set to 0.999, and the weight decay factor λ was set to 0.01.

To enhance optimization efficiency, gradient accumulation was implemented. This strategy aggregates gradients across multiple mini-batches before parameter updates, enabling larger effective batch sizes under limited GPU memory constraints.^[45]^ In this study, gradients were accumulated over 4 mini-batches per parameter update, balancing computational efficiency and convergence stability.

#### 2.5.7. Model evaluation and tuning

During training and validation, model performance was comprehensively evaluated using metrics including accuracy, sensitivity, precision, specificity, F1 score, and area under the receiver operating characteristic curve (AUC).^[46]^ To achieve continuous performance optimization, an intelligent hyperparameter tuning strategy combining grid search and Bayesian optimization was employed. This approach adaptively adjusted critical hyperparameters such as learning rate, weight decay coefficient, and batch size,^[47]^ significantly enhancing the model’s generalization capability across diverse data distributions. Learning rate (η), weight decay (λ), and batch size were dynamically calibrated to balance convergence stability and computational efficiency. Metrics were monitored across training/validation splits to prevent overfitting and ensure robustness in longitudinal predictions. This hybrid optimization framework provided a more reliable quantitative analysis foundation for capturing the complex associations between depressive symptom trajectories and cognitive decline risks.

## 3. Results

### 3.1. Demographic characteristics of participants

After preliminary screening, 2119 depression patients met the inclusion criteria. Three sliding window datasets were constructed based on these participants. Descriptive statistics were presented as frequency and percentage (n [%]) for categorical variables and mean ± standard deviation for continuous variables. The results are summarized in Table 1.

**Table 1:** Demographic characteristics of participants.

Categories	Variable		
	Wave 1 → Wave 2 (n = 932)	Wave 2 → Wave 3 (n = 1023)	Wave 3 → Wave 4 (n = 801)
Gender			
Male	486 (52.15%)	546 (53.37%)	420 (52.43%)
Female	446 (47.85%)	477 (46.63%)	381 (47.57%)
Age	66.11 ± 5.29	66.18 ± 5.30	66.32 ± 5.13
Education level			
Primary school or below	775 (83.15%)	841 (82.21%)	624 (77.90%)
Middle school	106 (11.37%)	113 (11.05%)	112 (13.98%)
High school or technical school	37 (3.97%)	56 (5.47%)	53 (6.62%)
College or above	14 (1.50%)	13 (1.27%)	12 (1.50%)
Residence			
Rural	606 (65.02%)	647 (63.25%)	461 (57.55%)
Urban	326 (34.98%)	376 (36.75%)	340 (42.45%)
Marital status			
Married	758 (81.33%)	855 (83.58%)	663 (82.77%)
Unmarried	8 (0.86%)	7 (0.68%)	8 (1.00%)
Divorced	14 (1.50%)	8 (0.78%)	3 (0.37%)
Widowed	152 (16.31%)	153 (14.97%)	127 (15.86%)

### 3.2. Model performance and comparisons

**Figure 3.**
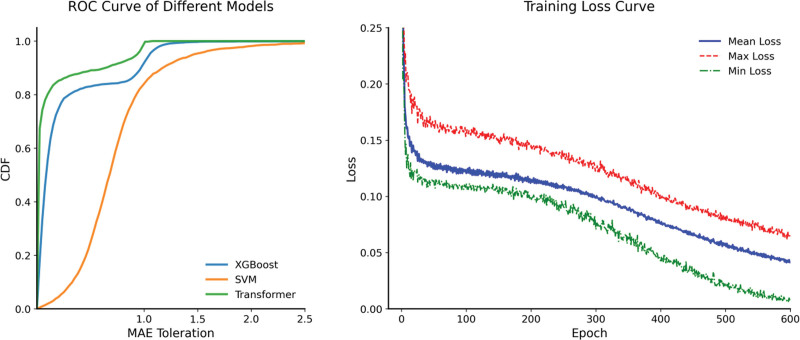
Performance comparison of XGBoost, SVM, and the optimized transformer model. SVM = support vector machine, XGBoost = Extreme Gradient Boosting.

By comparing the predictive performance of 3 machine learning models – Extreme Gradient Boosting (XGBoost), support vector machine (SVM), and the optimized transformer – for MCI secondary to depression, the results (Fig. 3 and Table 2) demonstrate the following.

**Table 2:** Model performance metrics.

Metrics	Training set			Test set		
	XGBoost	SVM	Fine-turned transformer	XGBoost	SVM	Fine-turned transformer
Accuracy	0.759	0.728	0.828	0.749	0.737	0.816
Sensitivity	0.779	0.772	0.812	0.774	0.757	0.801
Precision	0.784	0.733	0.890	0.761	0.732	0.892
Specificity	0.734	0.678	0.852	0.720	0.717	0.841
F1 score	0.782	0.752	0.849	0.768	0.744	0.844
AUC	0.812	0.787	0.845	0.807	0.776	0.851

AUC = area under the receiver operating characteristic curve, SVM = support vector machine, XGBoost = Extreme Gradient Boosting.

The optimized transformer model exhibited significant advantages across all metrics on the test set: Accuracy (0.816), Sensitivity (0.801), Precision (0.892), Specificity (0.841), F1 Score (0.844), and AUC (0.851). These results markedly outperformed XGBoost (AUC 0.807) and SVM (AUC 0.776), with precision and specificity improvements of 16.0% and 12.4%, respectively, over SVM. This superiority likely stems from the transformer’s self-attention mechanism, which effectively captures time-dependent associations between depressive symptoms and MCI onset. The dynamic relationship – such as fluctuations in CESD-10 subscores and longitudinal changes in delayed recall tasks – requires long-range dependency modeling, which traditional models (e.g., XGBoost’s tree structures, SVM’s kernel methods) struggle to resolve due to their limitations in capturing nonlinear interactions across temporal windows.^[48]^ In contrast, the transformer’s positional encoding and multi-head attention mechanisms explicitly model temporal feature relationships, enabling precise identification of early MCI risk patterns.

Notably, the transformer maintained high F1 scores and AUC values on the test set with minimal disparity from training set performance, reflecting strong generalization capability. This robustness likely arises from the MI strategy (minimizing noise from missing data) and sliding window segmentation (capturing localized temporal dependencies), which jointly reduce overfitting risks. In comparison, XGBoost showed slightly lower sensitivity (0.774) and AUC (0.807) on the test set, potentially due to tree model depth sensitivity to noise, SVM exhibited the smallest performance fluctuations between datasets but consistently lower metrics overall, highlighting its limited capacity for high-dimensional temporal data representation.

**Figure 4.**
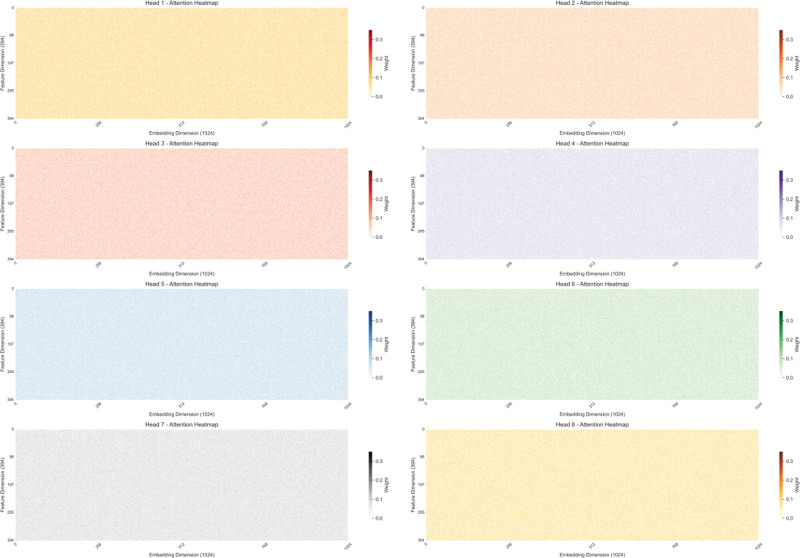
Multi-head attention heatmap of the optimized transformer model.

Figure 4 presents a multi-head attention heatmap of the fine-turned transformer model, where the *x*-axis denotes embedding dimensions and the *y*-axis represents features. Via a color-coding scheme, it illustrates the attention weight distribution between features and embedding dimensions as modeled by the transformer. Deeper colors correspond to higher attention weights, signaling a stronger focus by the model on those feature-embedding pairings. Through this mechanism, physicians may be able to prevent the risk of secondary MCI more timely through the representational state of some elderly patients with depression.

## 4. Discussion

### 4.1. Modelling analysis

With the intensification of global aging, the comorbidity of depression and cognitive impairment has become a major public health challenge. Early identification of geriatric MCI is critical for delaying Alzheimer disease progression, yet depressive symptoms – despite being significant prodromal markers – are often underestimated due to their high heterogeneity and complex dynamics. The transformer model, through its self-attention mechanism, explicitly quantifies temporal associations between depressive symptom trajectories and MCI risks. For instance, in the CESD-10 scale, cumulative increases in “loss of interest” and “sleep disturbances” scores across consecutive time windows (e.g., ≥2-point rise from Wave 1 to Wave 2) may be identified as high-risk signals by the model, with attention weights amplifying their predictive contributions. Additionally, the synergistic effect of longitudinal declines in delayed recall performance and depressive symptom fluctuations is captured as an early warning sign for MCI. Traditional models like XGBoost, constrained by the localized temporal modeling limitations of tree structures, fail to adequately characterize such cross-window dynamic patterns,^[49]^ resulting in lower sensitivity (0.774) and AUC (0.807) compared to the transformer.

By converting 4-wave longitudinal data into continuous temporal segments via a sliding window strategy (e.g., Wave 1 → Wave 2), the model enables dynamic risk prediction updates for MCI secondary to depression. This temporal framework allows clinicians to regularly update patients’ depression-cognition interaction trajectories and implement stepped intervention strategies. For example, a participant with a CESD-10 score increase from 11 to 13 points and a 3-point decline in delayed recall between Waves 1 and 2 would trigger the model’s multi-head attention mechanism to detect abrupt feature interactions, elevating MCI risk probability. Clinicians could then proactively initiate cognitive training or antidepressant therapy, avoiding reliance on overt clinical symptoms. In contrast, SVM’s rigid kernel-based representation of high-dimensional temporal data^[50]^ yields lower specificity (0.717) and precision (0.732), leading to higher false positive rates and reduced efficacy in distinguishing dynamic risk patterns.

The transformer’s high precision (0.892) and specificity (0.841) demonstrate its ability to accurately identify MCI high-risk populations driven by depressive symptoms, minimizing misclassifications caused by confounding factors such as age-related cognitive decline. For example, the model’s emphasis on patterns like “persistent depressive deterioration + declining delayed recall slopes” enables precise differentiation between depression-driven MCI and natural cognitive aging. This capability reduces unnecessary neuropsychological assessments or imaging studies (e.g., positron emission tomography scans), optimizing resource allocation and lowering healthcare costs. The robust AUC stability (≤0.7% disparity between training and test sets) further validates the sliding window strategy and MI method’s resilience to data fragmentation and noise.

While XGBoost and SVM perform adequately in static feature analysis, their limitations in temporal dynamic modeling hinder their applicability to depression-related MCI scenarios. XGBoost’s tree-splitting mechanism captures only local feature interactions (e.g., single-time-point CESD-10 scores) while neglecting cross-window cumulative effects (e.g., progressive depressive worsening combined with cognitive decline slopes), leading to a 0.5% sensitivity drop between training and test sets. SVM’s kernel-based approach struggles with high-dimensional temporal data, resulting in a significantly lower AUC (0.776) than the transformer, reflecting its inability to resolve complex combinations of depressive symptom “fluctuation frequency” and “amplitude changes.”

In the context of this study, the heatmap offers a glimpse into how the model processes temporal data to predict the risk of MCI secondary to depression. For instance, it reveals that fluctuations in certain features, such as scores reflecting “loss of interest” and “sleep disturbances,” are emphasized by the model when forecasting cognitive decline. This visual representation underscores the model’s capacity to identify critical feature interactions linked to MCI risk. Moreover, the heatmap elucidates the model’s feature selection and weight allocation mechanism. By fine-tuning attention weights, the model can pinpoint feature combinations highly correlated with MCI risk and embed them in a high-dimensional semantic space for analysis. This visual not only enhances the model’s interpretability but also provides clinicians with insights into the model’s reasoning, aiding in the optimization of intervention strategies. In practical terms, this visualization is instrumental for clarifying prediction outcomes and refining model parameters.

### 4.2. Clinical and public health implications

Leveraging CHARLS database multidimensionality and the model’s lightweight architecture, this method is deployable in primary care settings for cost-effective, large-scale geriatric mental health monitoring. The dynamic risk probabilities generated by the model provide a quantitative basis for aging-related health policies, enabling targeted resource allocation (e.g., strengthening mental health services in high-incidence regions). Furthermore, by integrating psychology, neuroscience, and AI, this study establishes a methodological paradigm for investigating interactions between psychiatric and neurodegenerative disorders, fostering interdisciplinary collaboration.

From a public health perspective, this model equips healthcare systems with a proactive tool to counter the escalating burden of cognitive impairment among elderly populations. By identifying high-risk individuals at early stages, it facilitates the implementation of cost-effective interventions, which could markedly reduce the progression to dementia and alleviate the strain on healthcare resources. The model’s deployment in community health centers can democratize access to specialized mental health monitoring, ensuring that even resource-constrained regions benefit from advanced predictive analytics.

For preventive medicine, this method underscores the importance of monitoring depressive symptoms as precursors to cognitive decline. It enables the formulation of evidence-based guidelines for early intervention, such as initiating cognitive training programs or psychotherapy for individuals exhibiting high-risk depression trajectories. This paradigm shift from reactive to preventive care could significantly lower the incidence of MCI and dementia in aging societies.

Subsequently, to validate the model’s generalizability, similar methodologies were applied to the Chinese Longitudinal Healthy Longevity Survey database.^[51]^ The model demonstrated superior performance, achieving an F1 score of 0.783 and an AUC of 0.821, which are the best among the 3 models. These results not only confirm the model’s robustness across diverse datasets but also reinforce its broad applicability and scalability in real-world settings. This cross-database validation ensures that the model can be reliably deployed across different populations, offering a versatile solution for combating cognitive decline in various public health contexts.

In the realm of public health and prevention, our model represents a significant advancement. By integrating data from the CHARLS database with advanced AI techniques, it provides a powerful tool for early detection and intervention in cognitive decline among the elderly. The model’s ability to generate dynamic risk probabilities allows for the timely identification of individuals at risk of MCI and dementia, enabling healthcare providers to implement targeted interventions before cognitive decline becomes irreversible. By providing a quantitative basis for aging-related health policies, it enables policymakers to make data-driven decisions regarding resource allocation and program development.

### 4.3. Limitations and future directions

Despite its strengths, this study has limitations: The sliding window strategy, while aligning with transformer’s fixed input requirements, may fragment long-term trends across windows. The final cohort of 2119 participants – diagnosed with MCI via statistical thresholds – may introduce heterogeneity biases.

Future research could explore: Temporal augmentation strategies or graph neural networks to model spatial propagation of cognitive decline. Multimodal data integration (e.g., fMRI functional connectivity,^[52]^ protein biomarkers^[53]^) to build cross-scale predictive frameworks for complex trajectory analysis.^[54,55]^

## 5. Conclusion

This study provides novel evidence for the pathological association between depressive symptoms and secondary MCI, guiding future mechanistic research. The heterogeneous distribution of attention weights may reflect distinct clinical subtypes of MCI, promoting the development of precision subtyping and diagnosis-treatment strategies.

Beyond methodological innovation in dynamic risk prediction for geriatric MCI, this research demonstrates profound multidimensional value across clinical practice (enabling proactive interventions), public health (optimizing resource allocation), technological outreach (providing AI-driven tools for large-scale screening), and scientific understanding (elucidating depression-cognition interactions).

Looking ahead, the integration of multimodal data (e.g., neuroimaging, biomarkers) and enhanced model interpretability will further empower this framework as a core tool for early warning and intervention in psychiatric and neurodegenerative diseases. By bridging AI analytics with clinical decision-making, this approach offers sustainable solutions for health management in aging societies worldwide, addressing the escalating challenges of cognitive decline in the global elderly population.

## Acknowledgments

The authors sincerely thank the China Health and Retirement Longitudinal Study (CHARLS) and the Chinese Longitudinal Healthy Longevity Survey (CLHLS) teams for providing access to the data used in this study. We also thank all survey participants for their valuable contributions. Their efforts have made important research on geriatric mental health and cognitive decline possible.

## Author contributions

**Data curation:** Yirui Chen, Siyi Kong.

**Formal analysis:** Siyi Kong.

**Investigation:** Yirui Chen, Siyi Kong, Junxing Chen.

**Methodology:** Yirui Chen, Tianyun Wang, Junzhi Zhang.

**Project administration:** Tianyun Wang, Junxing Chen, Kai Ma.

**Resources:** Ye Zhang.

**Software:** Junzhi Zhang, Ye Zhang.

**Supervision:** Junxing Chen, Kai Ma.

**Validation:** Kai Ma.

**Visualization:** Junzhi Zhang.

**Writing – original draft:** Yirui Chen, Tianyun Wang, Kai Ma, Junzhi Zhang.

**Writing – review & editing:** Yirui Chen, Ye Zhang, Mengyang Wang.
